# Fatal interstitial lung disease associated with oral erlotinib therapy for lung cancer

**DOI:** 10.1186/1471-2407-7-150

**Published:** 2007-08-05

**Authors:** Demosthenes Makris, Arnaud Scherpereel, Marie Christine Copin, Guillaume Colin, Luc Brun, Jean Jacques Lafitte, Charles Hugo Marquette

**Affiliations:** 1 Pulmonary and Thoracic Oncology Department, CHRU of Lille, Lille, France; 2 INSERM Unit 774, Institut Pasteur of Lille, Lille, France; 3 Pathology Department, CHRU of Lille, Lille, France; 4 Clinique des Maladies Respiratoires, Hôpital Albert Calmette, CHRU de Lille, 59037 Lille cedex

## Abstract

**Background:**

Erlotinib is a Human Epidermal Growth Factor Receptor Type 1/tyrosine kinase (EGFR) inhibitor which is used for non-small-cell lung cancer treatment. Despite that erlotinib is considered to have a favorable safety profile, adverse events such as interstitial lung disease (ILD) were reported in pivotal studies. The authors report the first histologically confirmed case of fatal ILD associated with erlotinib therapy.

**Case Presentation:**

The medical record of a patient who developed fatal ILD after receiving erlotinib treatment was reviewed to identify the cause of death and other factors potentially contributive to this adverse outcome. A 55-year-old smoker with no evidence of pre-existing interstitial disease developed bilateral ILD and respiratory failure which could be explained only as a toxicity of erlotinib. He had a history of stage IV left upper lobe squamous-cell carcinoma for which he had received three successive regimens of chemotherapy (ifosfamide plus gemcitabine, docetaxel, mitomycin plus navelbine), followed five months later by erlotinib. At initiation of erlotinib treatment there were no radiological signs suggestive of ILD disease or apparent clinical signs of respiratory distress. While the patient completed two months with erlotinib therapy he developed bilateral interstitial infiltrates; despite discontinuation of erlotinib he was admitted with respiratory failure two weeks later. Diagnostic work up for other causes of pneumonitis including infectious diseases, congestive cardiac failure and pulmonary infraction was negative. Empiric treatment with oxygene, corticosteroids and later with cyclophosphamide was ineffective and the patient progressively deteriorated and died. The clinical and post-mortem examination findings are presented and the possible association relationship between erlotinib induced ILD and previous chemotherapy is discussed.

**Conclusion:**

Physicians should be alert to the fact that erlotinib related ILD, although infrequent, is potential fatal. The association between selective EGFR-inhibitors and ILD should be further investigated.

## Background

Erlotinib (Tarceva^®^) is an Epidermal Growth Factor Receptor Type 1/tyrosine kinase (HER1/EGFR) inhibitor. The development of erlotinib in the treatment of advanced non-small-cell-lung cancer (NSCLC) raised a great enthusiasm among physicians. The initial safety and efficacy clinical studies showed some prolonged remissions and, in some cases, dramatic improvement in the quality of life in patients whose condition was no longer responding to standard chemotherapy. However, adverse events associated with erlotinib treatment, such as diarrhoea and rush, less often conjunctivitis and keratitis and rarely interstitial lung disease (ILD) have been observed [1]. We report the first histologically confirmed case of fatal ILD associated with erlotinib therapy.

## Case presentation

**Figure 1:**
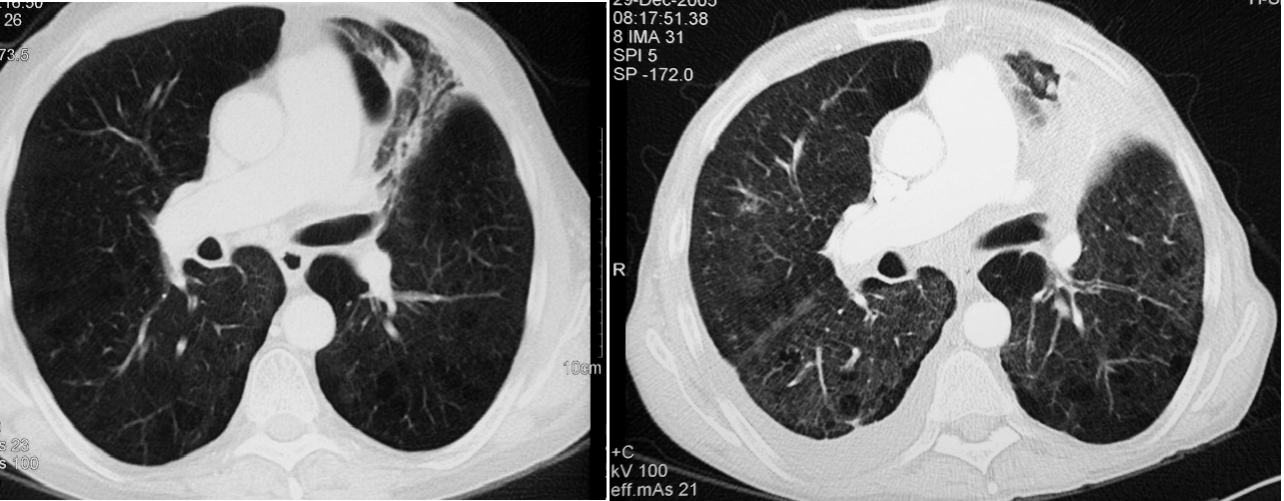
Chest CT scans before erlotinib therapy (left) and after two months of erlotinib therapy (right).

In January 2006, a 55-year-old smoker was admitted in our hospital with acute respiratory failure. The patient reported one-week of progressive exertional dyspnoea but denying chest pain, haemoptysis, increased cough or fever. He had a history of chronic obstructive pulmonary disease [baseline values: FEV_1 _= 900 ml (or 35 %predicted), FVC = 2.1 L (or 56 %predicted), FEV_1_/FVC(%predicted) = 45] while stage IV left upper lobe squamous-cell carcinoma was diagnosed fourteen months ago. He had received three successive regimens of chemotherapy (ifosfamide plus gemcitabine, between August and December 2004, docetaxel, between January 2005 and April 2005 and mitomycin plus vinorelbine tartrate, between April and May 2005), followed by erlotinib on October 2005. Two months later, while on erlotinib, he was restaged for his cancer. At that time clinical examination revealed minimal non productive cough and the presence of a facial exantheme which is a common side effect of erlotinib; the Karnofski index was 90% and oxygen saturation at rest was 96%. Computed Tomography showed no response of the primary tumour but revealed newly appearing bilateral diffuse ground-glass opacities (Figure 1); there was no evidence of pulmonary infraction/emboli. Because of reports of erlotinib associated ILD[1] the drug was withdrawn. However, two weeks later the patient was admitted with severe dyspnea. His temperature was 36.6°C, his blood pressure was 120/60 mmHg, pulse was 120 beats per minute. He was tachypnoeic with 30 breaths per minute. Arterial blood gasses at rest (FiO_2 _= 0.21) were: PaO_2 _43 mmHg and PaCO_2 _53 mmHg. Cardiovascular evaluation was normal with no evidence of significant jugular venous distension or peripheral oedema. Chest examination revealed bibasilar inspiratory crackles. Leucocyte cell count was 12/mm^3 ^with 67% neutrophils. All cultures and stains for infectious etiologies including common bacteria, fungi, pneumocystis, legionella, nocardia, viruses were negative. Sputum and gastric fluid culture proved negative for mycobacteria three weeks later. The patient was started on supplemental oxygen and iv. methylprednisolone (1 mg/Kg daily and then 3 g bolus therapy after one week) and empiric therapy was expanded one week later to include cyclophosphamide (500 mg). Despite transient clinical improvement, hypoxemia persisted and oxygen requirements increased. The patient progressively deteriorated and died three weeks later.

**Figure 2:**
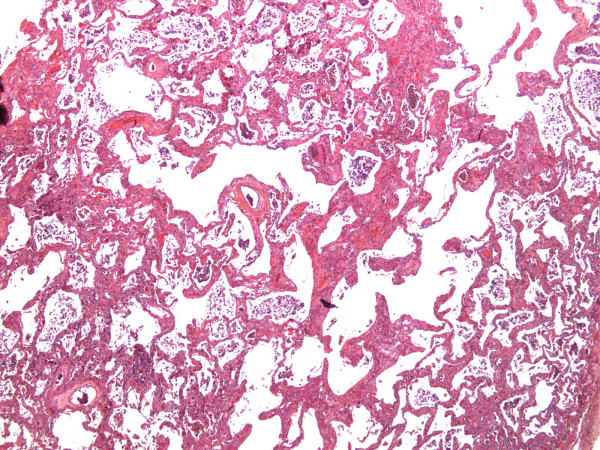
Diffuse alveolar septal thickening by fibrosis with enlargement of alveolar spaces (HES magnification × 25).

At autopsy, the heart was normal with no signs of globoid enlargement of atrials or ventricles, nor signs of cardiac ischemia. The lungs appeared enlarged. Macroscopic examination revealed a 6.5 cm upper left mass and a 1.5 cm upper right mass which at microscopic examination was proved to be a squamous cell carcinoma. Both lungs showed homogenous airspace consolidation predominating at the lower lobe of the right lung. Microscopic examination showed scattered lesions of organizing pneumonia and thickening of the vascular walls mainly in the peritumoral parenchyma, probably related to the effects of chemotherapy. At low magnification (Figure 2), alveolar spaces were enlarged and surrounded by patchy interstitial fibrosis distributed throughout the lungs, and lined by hyperplastic type II pneumocytes, suggestive of the organizing stage of a diffuse alveolar damage. Some thrombi were observed in small-sized pulmonary arterioles. At autopsy, there was no evidence of infection, carcinomatous lymphangitis or cardiogenic pulmonary oedema.

## Discussion

The present case demonstrated that erlotinib may be associated with fatal ILD. Erlotinib either caused or contributed significantly to the pulmonary damage, and thus this drug must be considered among the antineoplastic agents which can cause or contribute to interstitial pulmonary disease. This is important information since erlotinib is considered as a new promising oral chemotherapy agent in NSCLC, with a favourable safety profile[1]. To our knowledge, this is the first histologically confirmed case of fatal ILD, associated with erlotinib treatment.

Tammaro et al[2] reported a case of ILD following erlotinib diagnosed on basis of clinical and radiological criteria, without histological confirmation. In that case, a favourable outcome was observed with steroids. Marked improvement in symptoms and radiologic signs was noted when the patient had completed one month therapy with prednisolone. However, severe pulmonary adverse events, including fatalities had been reported with another EGFR-inhibitor (gefinitib-Iressa^®^)[3]. Thus, FDA advises that erlotinib should be suspended pending evaluation, on the appearance of new pulmonary symptoms and discontinued upon confirmation of ILD[1]. The present case confirms that the outcome of erlotinib related ILD may be extremely unfavourable[1]. The onset of the disease was indolent and, once patent, progressed until death despite withdrawn of erlotinib and administration of empiric treatment.

The diagnosis of drug-induced ILD relies on typical radiologic features and exclusion of other potential causes. In our case radiologic signs of ILD were apparent within the first two months of erlotinib treatment in accordance with the report of previous studies [1-3]. The diagnosis of erlotinib induced pulmonary injury was made by excluding other potential causes such as congestive heart failure, infections, or lymphangitic carcinomatosis. Thoracic radiation or pre-existing interstitial disease that have been reported to contribute to ILD secondary to EGFR-inhibitors[3] were not present in our case. It should be underlined here that patients on erlotinib have usually already received treatment with various antineoplastic agents. Previous chemotherapy was reported as a predisposing factor for gefinitib related ILD[3]. Interstitial pneumonitis, acute hypersensitivity pneumonitis, acute permeability edema with or without acute respiratory distress syndrome were described for gemcitabine[4], mitomycin[5], vinorelbine tartrate[6], docetaxel[7], or ifosfamide[8] and occur usually early after the administration of chemotherapy. Whether erlotinib can induce pulmonary toxicity by a mechanism related to its unique properties or contributes significantly to pulmonary toxicity induced by prior chemotherapy, it remains to be investigated.

The incidence of ILD was less than 1% in erlotinib pivotal trials[1]. However, the incidence could be higher since ILD diagnosis requires diagnostic work up that may not be always feasible in patients with advanced non-small-lung cancer and/or co-morbidities. Notably, the frequency of ILD associated to gefinitib appeared to be higher in case series[3] and observational studies[9] than in pivotal trials[10]. Thus, clinical studies would be helpful to confirm whether the incidence of erlotinib related ILD is as low as 0.6% especially considering the potential danger imminent in erlotinib related ILD.

## Conclusion

In conclusion, physicians should be alert to the fact that ILD following erlotinib, although infrequent, is potentially fatal. This observation reinforces FDA advice regarding the necessary alertness of physicians to detect early radiographic signs of ILD in patients on erlotinib. The association between selective EGFR-inhibitors and ILD should be further investigated.

## Competing interests

The author(s) declare that they have no competing interests.

## Authors' contributions

DM drafted the manuscript and participated in data collection. AS participated in the design and coordination of the case-study and revised the article for important intellectual content. MCC carried out the histological examination. GC participated in the preparation of the manuscript and in the data collection. LB carried out autopsy and participated in the histological examination. JJL participated in the preparation of this article by revising it for important intellectual content. CHM coordinated the study and revised the article for important intellectual content. All authors read and approved the final manuscript.

## Pre-publication history

The pre-publication history for this paper can be accessed here:


